# Dried Blood Spots for Global Health Diagnostics and Surveillance: Opportunities and Challenges

**DOI:** 10.4269/ajtmh.17-0889

**Published:** 2018-07-02

**Authors:** Mark D. Lim

**Affiliations:** Global Health Division, Bill & Melinda Gates Foundation, Seattle, Washington

## Abstract

There is increasing interest in using dried blood spot (DBS) cards to extend the reach of global health and disease surveillance programs to hard-to-reach populations. Conceptually, DBS offers a cost-effective solution for multiple use cases by simplifying logistics for collecting, preserving, and transporting blood specimens in settings with minimal infrastructure. This review describes methods to determine both the reliability of DBS-based bioanalysis for a defined use case and the optimal conditions that minimize pre-analytical sources of data variability. Examples by the newborn screening, drug development, and global health communities are provided in this review of published literature. Sources of variability are linked in most cases, emphasizing the importance of field-to-laboratory standard operating procedures that are evidence based and consider both stability and efficiency of recovery for a specified analyte in defining the type of DBS card, accessories, handling procedures, and storage conditions. Also included in this review are reports where DBS was determined to not be feasible because of technology limitations or physiological properties of a targeted analyte.

## INTRODUCTION

Most diagnostics and surveillance programs rely on measurements from an individual’s blood specimen to guide a clinical or public health decision. To minimize pre-analytical sources of data variability, processes for venipuncture collection are standardized through devices such as analyte-specific blood collection tubes and evidence-based best practices, guidelines, and protocols.^1,2^ Global health settings often lack infrastructure for quality-assured venipuncture,^3^ sparking significant interest in the use of dried blood spot (DBS) cards as a universal solution.^4–11^ The intent of this review is to underscore the need to assess the reliability of DBS-based bioanalysis in context to a specific biomarker and envisioned field-to-laboratory workflow, before applying this technology into a remote health or surveillance program.

Compared with venipuncture, the value proposition of DBS is simplified logistics for remote sampling through:

- Reduced workforce requirements
- Smaller volumes of blood and components (plasma and serum)
- Direct heelprick/fingerprick-to-DBS or indirect capillary-to-DBS deposition of blood
- Collection of nonblood biofluids such as saliva
- Simplified transport, shipment, and disposal
- Simplified biobanking for retrospective analysis

Commercially available DBS cards are not designed for the minimally resourced environments typical of remote health settings and instead are primarily used in newborn screening and preclinical drug development by highly proficient personnel within controlled clinical and laboratory environments. For instance, most DBS are susceptible to contamination by the user, patient, environment, insects, equipment, or contact with other cards. Health-care workers also have a risk of exposure to potentially infectious agents until blood is dried and contained in secure packaging. Most of these risks can be mitigated through standard operating procedures and accessories, but the impact of these variables on data quality needs to be assessed through careful studies simulating the pre-analytical workflow, starting with specimen acquisition to DBS preparation for analysis. Readers are advised to review the comprehensive review of mass spectrometry (MS) methods^12^ and the collection of reports compiled by Li and Lee discussing various use cases, techniques, and technologies for DBS-based bioanalysis.^13^

Two primary global health applications envision that the use of DBS can extend either health-care services or research and surveillance studies into harder-to-reach populations. The clinical scenario aims to measure health-related diagnostic data to stratify at-risk individuals for additional confirmatory testing or to guide individual- or population-level treatment decisions. The other scenario aims to extend epidemiological surveillance that monitors population-level transmission of infection or tracks emerging or recrudescing disease. Both scenarios rely on tools that provide high-sensitivity analysis of individual samples to minimize the risk of missed positive cases, particularly in geographies where loss to follow-up remains a significant challenge. In other words, for both scenarios, false-negative test results typically have higher consequences for these programs than false-positive test results if there is an opportunity to further confirm the clinical or epidemiological status of test-positive individuals or populations.

The weakest link for sensitivity within a bioanalytical workflow is the quality of the specimen.^2^ The concept of DBS is appealing; however, these broad remote-sampling aspirations should consider the extensive literature evaluating the reliability of DBS for high-sensitivity analysis of specific biomarkers. In most instances, quantitative studies have demonstrated the feasibility of DBS if standardized collection and laboratory protocols are followed.^12,14–18^ However, there are examples where DBS fails to provide reliable results and this review includes a sample of these reports.

## BACKGROUND

The concept of depositing fingerprick-derived blood on laboratory filter paper, the precursor of DBS, was first described in the 1860s for glucose measurements^15^ and in the 1960s for screening metabolic disorders in newborns using heelprick-derived blood.^19^ One of the popular DBS formats is the Whatman 903 card, which is composed of cotton-based filter paper within a rigid cardboard frame for handling and labeling. The paper is ink-printed with five half-inch circles that direct the user to the location for depositing a specimen. Blood-deposited cards are typically dried in an open environment by suspension in ambient air or under forced circulation in a laboratory or hospital.^20^ Dried blood spots are often stored for transport in a sealed bag with desiccant and archived under refrigerated or frozen conditions.^15,16^ Portions of the dried spot are “punched” out with a regular hole puncher or scissors, specialized DBS punchers and protocols are both available to reduce risk of contamination by card-to-card carryover.^21–24^ The whole spot can also be used if there are no plans to re-analyze or archive the specimen.

**Figure 1.**
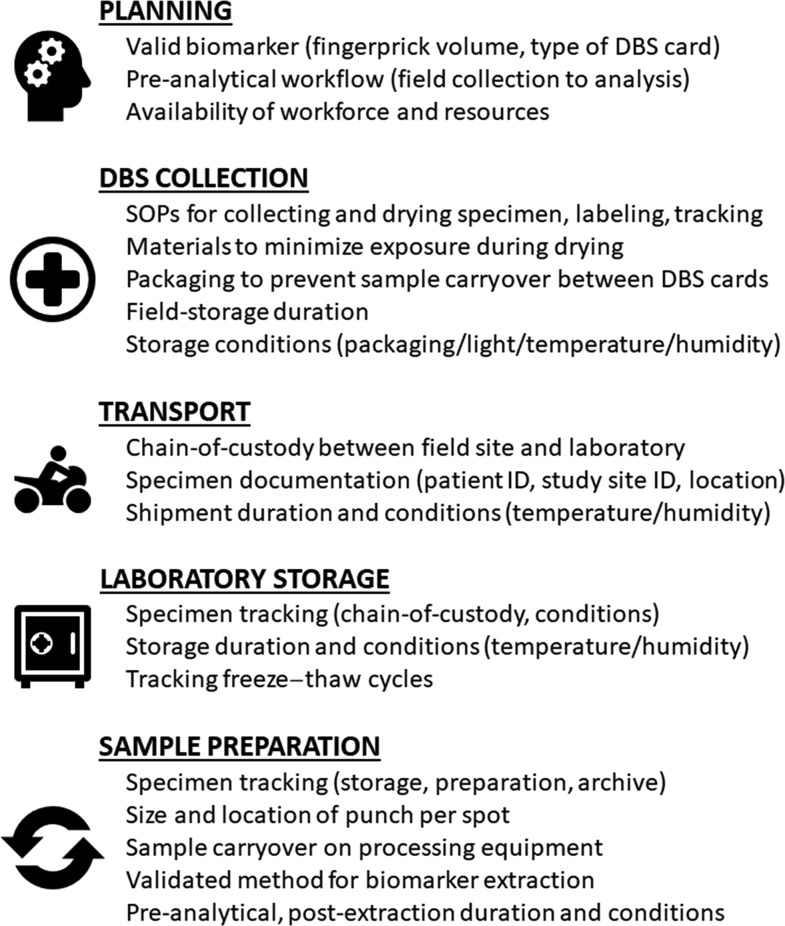
Non-exhaustive list of pre-analytical considerations when using dried blood spot (DBS) in field settings.

The panel of diseases screened by newborn programs has significantly expanded since Guthrie’s first application of DBS^25^ with interest to use this technology in global health strategies.^4,10,11,26–31^ Given the implications of test results on treatment decisions or public health resources, published protocols and guidelines aiming to minimize the risk of pre-analytical variability are regularly evaluated and updated.^9,16,20,32–34^ Some assessments have found that a diagnostic cutoff determining one decision over another may be dependent on the type of platform used to analyze a DBS-derived specimen, such as those using human immunodeficiency virus (HIV) viral load measurements to determine treatment effectiveness^27,35–40^ or polymerase chain reaction (PCR) analysis of malarial DNA.^41^ These findings stress the importance of assessing and mitigating sources of data variability within a complete field-to-laboratory pre-analytical workflow, starting with the type of DBS and the platform used for downstream analysis of a specific biomarker (Figure 1).

The drug development sector is another early adopter, envisioning that DBS provides a simplified and cost-effective approach for measuring drug metabolites and toxicology biomarkers in preclinical animal studies.^42–44^ This community published most of the quantitative evaluations in an effort to support claims on the equivalency of DBS-based data to data from venipuncture.^18,45–47^ Recent efforts to evaluate the feasibility of DBS for remote clinical trials have also been met with successes and challenges.^30,48–56^ One common conclusion from the newborn screening and drug development communities is the importance of storing DBS cards in refrigerated and desiccated conditions as soon as the specimen is dried to reduce data variability. The impact of these mitigation measures is dependent on the individual stability and physiological profiles of a specific analyte with frozen biobanking conditions still failing to provide sufficient stabilization over extended periods of time for many analytes. Some of the literature reviews summarizing the feasibility of DBS in global health applications include hepatitis B and C,^8,29,57–61^ HIV,^8,27,62–66^ and malaria.^30^

Evaluations of discordant DBS results identified sources of variability that include

- Interindividual differences, with a particular emphasis on hematocrit (Hct)
- Differences in analyte abundance between capillary and venous systems
- Type of DBS card
- Heterogeneity within a single dried spot, particularly if only a portion is used for analysis
- Storage conditions during transport and archive
- Sample preparation methods

It is important to note that sources of variability are often interconnected. As discussed later, Hct and homogeneity of a dried spot are linked and the impact of these variables on test results might also depend on the type of DBS card and the chemical and physical properties of an analyte. The possibility of multiparametric sources of variability reinforces the need for analyte-specific quantitative evaluations that define the conditions, processes, and tools that will be locked down as described within a standard operating procedure and reinforced through quality assessments during and after implementation.

Few improvements to the paper-based backbone of DBS have been pursued other than the development of cellulose-based formats to enhance extraction of some classes of analytes or addition of embedded chemicals to increase nucleic acid stability. There are recent efforts to improve the field-ability of DBS through accessories that reduce the risk of cross contamination or improve desiccation of the collected sample and Table 1 provides examples of commercially available technologies.

**Table 1:** Examples of commercially available dried blood spot cards and accessories (no endorsements should be implied by their listing in the table)

Brand name	Use case	Manufacturer
GE/Whatman FTA DMPK-A (Refs. 89,149)	Cellulose-based paper	GE Healthcare
	DNA stabilization with smaller blood volumes (10–20 μL) compared with FTA/DMPK-B	
	Impregnated with radical inhibitors [sodium dodecyl sulfate, tris (hydroxymethyl)aminomethane]	
	Cell lysis and protein denaturation on contact	
GE/Whatman FTA DMPK-B (Refs. 89,149)	Cellulose-based paper	GE Healthcare
	Similar to FTA DMPK-A, but blood spot area is 20% larger	
	Impregnated with chaotropic agents (guanidium thiocyanate)	
	Cell lysis and protein denaturation on contact	
GE/Whatman FTA DMPK-C (Refs. 89,149)	Cotton-based paper	GE Healthcare
	No impregnated stabilization chemicals, claimed to be suitable for protein-based analysis	
GE/Whatman 903 sample collection cards (Refs. 89,149)	Cotton-based paper	GE Healthcare
	Manufactured in slightly acidic environment, compared with FTA DMPK-C	
	No impregnated stabilization chemicals	
PerkinElmer/Ahlstrom 226	Cotton-based, no impregnated stabilization chemicals	PerkinElmer
HemaSpot-HF blood collection device	Collection, storage, and aliquots of finger-stick blood	Spot on Sciences
HemaSpot-SE blood separation device	Separation of serum/plasma from finger-stick blood	Spot on Sciences
AdvanceDx100	Separation of serum from finger-stick blood	Advance Dx
Mitra microsampler	Fixed-volume blood collection (volumetric absorptive microsampling)	Neoteryx

DMPK = drug metabolism and pharmacokinetics; FTA = Flinders Technology Associates.

## VARIABLES

### Hematocrit.

The predominant source of interindividual variability studied in the DBS literature is Hct, a measurement representing the percentage of red blood cells in a known volume of blood.^50,67^ These clinical measurements are used to determine if a patient has anemia and can vary by age, gender, health, living environment, and nutritional status. From a chemical and materials perspective, a blood sample with an elevated Hct would have increased viscosity because of the higher percentage of red blood cells. As described in several reports, a viscous blood drop would have a less homogenous spread across a piece of filter paper compared with a sample with lower Hct.^56,67–70^ These physical dynamics introduce at least three potential interlinked sources of variability: material composition of DBS, location of the “punch” within a heterogenous spot, and extraction method. The latter is likely to be dependent on the physical density of the dried blood as determined in part by an individual’s Hct. De Kesel et al.^68^ provide a comprehensive review of different drug development studies evaluating the implications of Hct on DBS-derived data. Across these reports, the degree of variability from different Hct was dependent on the properties of a specific analyte and the type of DBS used, complicating possibilities for a simple Hct correction factor.

The impact of Hct on newborn screening results was evaluated by the U.S. Centers for Disease Control and Prevention.^71^ Similar to a study conducted by a U.K. newborn screening network,^72^ Hall et al. observed that the total volume of dried blood within each spot increased with higher Hct. The opposite effect was noted for dried serum where higher Hct was associated with lower per-punch volumes of serum. The physical diameter of the DBS was related to the total volume of blood but not significantly affected by Hct. In addition, per-punch volumes of whole blood, red blood cells, and serum were differentially impacted by Hct level, potentially affecting reliability of test results if targeted analytes naturally partition between these blood components.

In addition to physical dynamics, several studies evaluated the impact of Hct on the measurement of individual markers. For instance, most newborn screening panels include congenital hypothyroidism, a condition with no visible symptoms and without immediate treatment that could result in mental retardation. The risk of a newborn developing this condition is determined by quantitation of thyroid-stimulating hormone (TSH, thyrotropin), a peptide of approximately 200 amino acids. Butler et al.^73^ reported that an increase in Hct resulted in an artificial and clinically significant decrease in TSH concentration, using DBS samples collected as part of a newborn screening program. Although the opposite effect (decreased Hct, increased TSH) was also observed, false-negative test results caused by an Hct-influenced decrease of TSH have greater health implications, particularly if this measurement is the basis of a primary screen.

Unfortunately, there is no direct method to determine Hct directly from a DBS sample. As mentioned earlier, the diameter of the total blood spot has been shown to have little change over a range of Hct^71^ and an endogenous or exogenous marker has yet to be validated.^56,74–76^ One suggestion is to control the total volume of blood deposited on a DBS using a volumetric capillary or fixed-volume accessory.^77–79^ Another possible mitigation is to ensure that the complete dried spot, including colorless plasma, is used for analysis rather an aliquoted punch. If the whole DBS circle is not used, location of punch relative to the whole spot should remain consistent along with annotation of an individual’s Hct.^22,70^ These additional procedures and accessories to mitigate this source of data variability increase logistical requirements in the field and laboratory, representing one of many trade-offs that should be assessed before scaling the use of DBS within remote health and surveillance strategies.

### Analyte abundance in venous versus capillary blood.

There are several reports describing a difference in the natural abundance of biomarkers between capillary and venous blood,^80–82^ some of which are described in Table 2. For low-abundant analytes, the impact of this variability on test results is likely to be amplified, given that smaller volumes of blood collected in DBS increase the probability of false-negative detection.^83,84^ Unlike venous blood draw, there is also a higher risk for inconsistent volumes with static fingerprick- or heelprick-drawn blood; “milking” the skin puncture to increase volume through direct pressure also increases risk for hemoconcentration.

**Table 2:** Examples of reports investigating physiological differences in biomarker abundance between capillary and venous blood

Disease/infection	Type of biomarker	Difference in capillary blood, compared with venous blood	Ref.
Zika	Viral load	Higher in capillary blood	150
Anemia	Hemoglobin	Higher in capillary blood	151
Hematological assessments	Hematocrit	Higher in capillary blood	152
	Erythrocytes	Higher in capillary blood	
	Thrombocytes	Lower in capillary blood	
Malaria	G6PD	Differences attributed to type of analytical platform	153
Malaria	Drug metabolite (tafenoquine)	No significant difference	154
Malaria	Drug metabolite (piperaquine)	Higher in capillary blood	155
HIV	CD4 count	Lower in capillary blood using PIMA platform	156
HIV	Viral load	No significant difference	157
Hepatitis C	Viral load	No significant difference	158
Dengue-1	Viral load	Lower in capillary blood	159

G6PD = glucose-6-phosphate dehydrogenase.

Different hemoglobin concentrations were measured when fingerprick blood was extracted from either the left or right hand of the same individual.^85^ Bond and Richards-Kortum also reported higher degrees of variability in hemoglobin, white blood cell, and platelet count measurements from fingerprick-derived blood compared with venous-derived blood.^84^ In that study, collecting larger volumes of blood did not reduce the difference in total variability between capillary- and venous-derived blood (i.e., increased fingerprick volumes did not align the magnitude of variability with venous-derived blood). The authors also reported different degrees of drop-to-drop variability between patients, sharing that these results indicate interindividual physiological differences in the abundance of these analytes between the two circulatory pathways. Adding to the complexity, Hct levels were reportedly higher in capillary blood compared with venous blood in neonatal and young infants.^86^

As demonstrated by these examples, partitioning between venous and capillary circulation is likely analyte specific and cannot be simply rationalized by smaller diameters of peripheral capillaries or mitigated by larger volumes of collected blood. Higher concentrations of some analytes in capillary blood can be attributed to hemoconcentration effects or, alternatively, lower concentrations may be due to the presence of extracellular fluid.^86,87^ This physiological basis for biomarker partitioning needs to be considered in guiding the clinical relevance of bioanalytical data, particularly if reported clinical correlations are based on a different source or component of blood.^88^ Assessments of analytical and clinical equivalence for any biomarker measured by capillary- and venous-derived samples should be conducted before implementing DBS within a research study or clinical workflow.

### Type of DBS.

As shown in Table 1, different DBS formats are commercially available with some claiming improved extraction efficiency or stability for specific classes of analytes. The appropriate selection of the card should be based on the analyte properties and its stabilization requirements, extraction efficiency, and method of analysis. Basic DBS formats include the paper-based Whatman 903 and Ahlstrom 226 cards, and specialized cards include the Flinders Technology Associates (FTA) Drug Metabolism and Pharmacokinetics (DMPK) cards that are impregnated with cell lysis and analyte-stabilizing materials. Flinders Technology Associates DMPK-A cards contain sodium dodecyl sulfate and tris- (hydroxymethyl)aminomethane, and FTA DMPK-B cards contain guanidium thiocyanate.^89^ Some of these chemicals have been reported to leach from DBS during sample preparation and potentially interfere with some analytical platforms through a “matrix effect,” a risk that has been evaluated extensively by the drug development community.^90,91^

Analyte- and DBS-specific data variability was found between different FTA DMPK cards used for the measurement of small-molecule drugs in whole blood by liquid chromatography/MS.^12,89^ In addition to the matrix effect caused by impregnated chemicals, the type of paper can also influence spreading dynamics of blood across the DBS, as mentioned earlier.^56,92,93^ Distribution of antibodies across a DBS spot was found to be heterogenous and not predictable with authors suggesting that greater than 15% variability between different punches of the same spot.^41^ The impact of Hct and concentration on the recovery of small drug analytes were also dependent on the type of DBS.^94^

Of relevance to global health applications, the variability of HIV viral load measurements was evaluated in three different types of DBS^95,96^ and across different RNA analysis platforms.^97^ These studies observed variable genotyping efficiencies and drug susceptibility test results from samples derived from different DBS cards; some of the authors suggest that storage conditions might mitigate paper-dependent bias. Dried blood spot–based samples were also found to result in test results that overestimate HIV incidence when compared with test results from plasma samples.^98^

The recovery and stability of malaria-related histidine-rich protein 2 (HRP2)^99^ and mRNA were also reported to be dependent on the type of DBS.^100,101^ In a report by Miguel et al.,^102^ none of the three commercially available reagents were able to reliably extract DNA associated with *Plasmodium falciparum* or *Plasmodium vivax* infection from blood stored on cotton-based filter paper, although others have shown a dependence on the type of DBS.^41,103^ The use of cards designed to preserve nucleic acids was found to provide sufficient stability for detecting single-species malaria infection but failed to diagnose individuals with mixed *P. vivax/falciparum* infections.^104^ Addressing these limitations, a method for the simultaneous extraction of nucleic acids indicating *P. vivax* and *P. falciparum* infections was developed and field-evaluated with slight differences in analytical performance reported between two types of DBS cards.^105^ Different sample preparation methods resulted in discordant results when using PCR to detect malaria parasites, particularly if only a single aliquot/punch was used.^41^ These reports reinforce the need to carefully select the type of DBS card with criteria based on the properties of a specific biomarker, method for analysis, and physiochemical interactions with the card materials, in context of optimized stabilization and sample preparation.

## STORAGE CONDITIONS

The impact of post-collection storage conditions on data quality has been extensively evaluated by multiple communities, focused on the type of card, time, temperature, humidity, and storage methods.^24,106–108^ These conditions include storage in the field, conditions during transport, storage before sample preparation, and longer term biobanking. The effects of these parameters are often dependent on the properties of the analyte and DBS, with a general recommendation that many of these impacts can be mitigated by storing dried cards in desiccated and frozen conditions as soon as possible.^109–112^

As mentioned earlier, biobanking in frozen conditions can fail to stabilize some analytes over extended period. For example, standard protocol for HIV viral load measurements calls for the immediate storage of DBS to less than −20°C or no longer than 14 days at ambient temperature. Even if stored at −20°C, DBS cards are only reliable if these measurements are made within 2 years.^16^ Similar recommendations are also described for storing DBS used in newborn screening and other clinical tests.^32,111,113,114^

Temperature and humidity conditions directly affect the ability to detect specific amino acids and metabolites routinely measured for newborn screening.^115,116^ Gene transcriptomics analysis of newborn DBS was more consistent if samples were stored at temperatures less than −20°C immediately after specimen acquisition,^111^ with time and temperature imparting various degrees of degradation for specific mRNA profiling targets and housekeeping genes.^112^ Lower temperatures is not the solution for all analytes; three polyunsaturated fatty acids used to screen newborns for neural development and visual function were found to have significant degradation after 10 days of storage at −28°C, with a high degree of intraindividual variability, when measured from umbilical blood dried on Ahlstrom 226 cards.^117–119^

For function-based bioanalysis, DBS storage temperatures greater than 4°C reduced the activity of all five enzymes measured to diagnose newborns at risk of lysosomal storage disorders, with the degree of variability dependent on the properties of a specific enzyme.^120–123^ Quantitative measurements of glucose-6-phosphate dehydrogenase (G6PD) are used by both malaria and newborn screening programs to identify individuals deficient of this essential enzyme. Two studies showed that temperature and humidity impacted quantitative measurements of G6PD activity, a source of variability that can be mitigated if DBS was stored under desiccated and refrigerated conditions.^124,125^

There is emerging literature describing the pre-analytical impact of DBS storage conditions on bioanalytical test results for other diseases of global health interest such as hepatitis B and C^29,58–61,126–130^ and dengue.^131,132^ DNA measurements of *P. falciparum* were affected by type of DBS, drying time, and humidity, with an overall inferior sensitivity compared with frozen whole blood.^31,133–137^ Incomplete drying, storage temperature, and humidity affected measurements of malaria gametocyte mRNA more significantly on samples derived from FTA DBS compared with Whatman 903 cards.^100,138^ Type of DBS and storage temperature and humidity affected stability and recovery of antibodies for malarial serological surveys.^139^ For HRP2 measurements on Whatman 903 cards, storage at temperatures less than −20°C significantly reduced the variability of test results from archived samples.^99^

For HIV drug resistance testing, HIV-1 nucleic acids were stable in DBS if stored in desiccated conditions at temperatures less than 4°C and were not recoverable if stored at 37°C under high humidity.^36,140–144^ Another report found that the rate of nucleic acid degradation because of storage conditions was dependent on a patient’s total viral load and preservation as dried blood or plasma.^143,145^ Similar to the malaria studies, drying time and handling of the specimen before biobanking affected the stability of HIV-1 RNA.^146,147^

Storage environment is not just one variable and includes temperature, humidity, and time within field, transport, and laboratory settings, all in context to the stability of a specific analyte. In many instances, the type of DBS was an important consideration. Storage procedures and conditions optimizing the stability of one biomarker are likely not optimal for a different analyte. This consideration is important for those developing multiplexed detection of a panel of analytes as trade-offs in analytical performance are likely and should be evaluated in the construction of a rigorous standard operating procedure.

## DISCUSSION

Dried blood spot offers several logistical advantages for remote health and surveillance programs, particularly for screening and surveying hard-to-reach populations. For many of these tests, a highly sensitive biomarker analysis is important for reducing the risk of missed positive cases. Analytical sensitivity not only includes the performance of a downstream platform but also the pre-analytical workflow that starts with the collection of a specimen from an individual. Quality assurance should not be the compromise of simplified logistics if incorrect test results have significant health implications or result in unnecessary expenditure of research and programmatic resources.

Although this review focused on evaluations of validated biomarkers, there is also significant interest in the use of DBS for biomarker discovery. Given current challenges of biomarker validation,^80,148^ DBS introduces interlinked sources of data variability that should be considered in any experimental design and statistical plan. As described in this review, significant effort is required to determine optimal conditions for specific analytes making broad standard operating procedures in the absence of an identified analyte overly simplistic. Field-collected DBS should be used sparingly in biomarker research or, at-minimum, in parallel with quality-assured venipuncture.

Several opportunities for improving the technology behind DBS should consider trade-offs with roll-to-roll DBS manufacturing processes,^149^ lower per-card cost, and simplified implementation logistics. In addition to direct measurements of Hct from DBS, there still lacks methods to determine the total volume of blood deposited on a card in the absence of a volumetric accessory. Simple field-appropriate technologies are also needed to control specimen drying and maintain desiccation of a blood spot on various types of DBS until storage in controlled conditions. Technologies that prevent contamination from other DBS cards, instrumentation, or environment would also be beneficial for the community.

Broad lessons learned include the importance of evaluating the physiological, chemical, and physical properties of each analyte in context to a conceptual pre-analytical workflow that includes DBS type, collection methods, and storage conditions. The newborn screening and drug development communities, in addition to an emerging community of global health researchers, continue to build on literature evaluating the reliability of DBS. These reports provide a foundation of methods for validating of DBS-based bioanalysis and for defining standardized procedures that ensure quality and reproducible data.
